# Synthesis of Bi_2_WO_6_/Na-bentonite composites for photocatalytic oxidation of arsenic(iii) under simulated sunlight

**DOI:** 10.1039/c9ra06181a

**Published:** 2019-09-19

**Authors:** Quancheng Yang, Yunxiang Dai, Zijian Huang, Jing Zhang, Ming Zeng, Changsheng Shi

**Affiliations:** School of Chemical and Environmental Engineering, China University of Mining and Technology Beijing 100083 P. R. China; Key Laboratory of Environmental Nano-Technology and Health Effect, Research Center for Eco-Environmental Sciences, Chinese Academy of Sciences Beijing 100085 P. R. China jingzhang@rcees.ac.cn; National Engineering Laboratory for VOCs Pollution Control Materials & Technology, University of Chinese Academy of Sciences Beijing 101408 P. R. China; Department of Environmental Engineering, North China Institute of Science and Technology Beijing 101601 P. R. China northinstitute@yeah.net

## Abstract

Novel Bi_2_WO_6_/bentonite (denoted as BWO/BENT) composites were prepared *via* a typical hydrothermal process and employed for the photocatalytic oxidation of arsenic(iii) (As(iii)). The properties of the prepared samples were characterized through X-ray diffraction, transmission and scanning electron microscopy, UV-visible diffuse reflectance spectroscopy, X-ray photoelectron spectroscopy, and photoluminescence spectroscopy. Effects of the BENT ratio on the As(iii) removal were explored under simulated sunlight, and the best photocatalytic effect was observed for the composite with BWO : BENT = 7 : 3 w/w. Compared with the pure BWO, the BWO/BENT composites exhibited an improved photocatalytic ability in the removal of As(iii), which was mainly ascribed to the enlarged specific surface area and the suppressed electron–hole recombination by the incorporated BENT. Furthermore, photo-generated holes (h^+^) and superoxide radicals ·O_2_^−^ were confirmed to be the major contributors to the oxidation of As(iii), and an associated mechanism of photocatalytic oxidation of As(iii) over BWO/BENT composites was proposed.

## Abstract 2

Novel Bi_2_WO_6_/bentonite (denoted as BWO/BENT) composites were prepared *via* a typical hydrothermal process and employed for the photocatalytic oxidation of arsenic(iii) (As(iii)).

## Graphical abstract

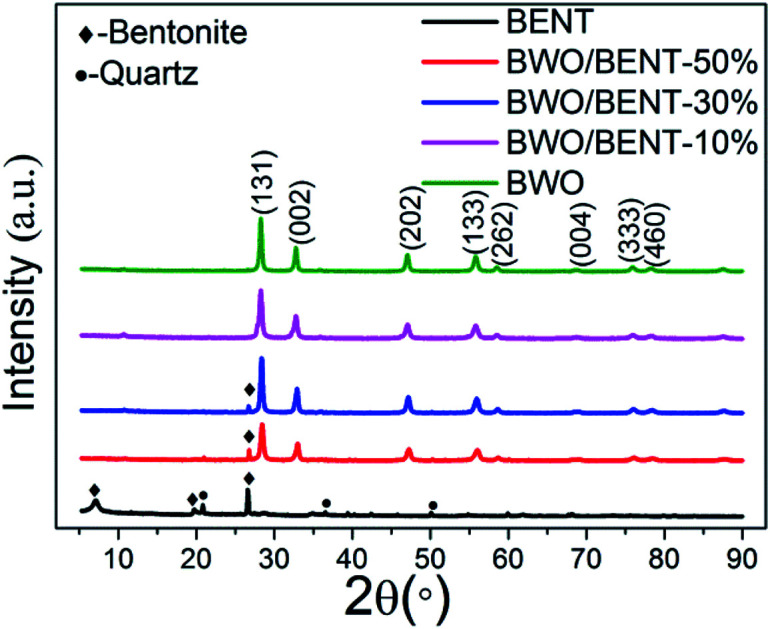

## 1. Introduction

Arsenic is an element widely applied in chemical, metallurgical, pharmaceutical, and other manufacturing industries.^1^ Carcinogenic arsenic contamination of groundwater and soil, induced by geogenic processes and anthropogenic activities, is a serious threat to millions of people and other living organisms around the world.^2^ Prolonged exposure to an arsenic polluted environment can significantly increase the risk of various kinds of cancers. To minimize this harm to health, the World Health Organization (WHO) stipulated the permitted arsenic concentration in drinking water to be 10 μg L^−1^.^3,4^ The toxicity of arsenic is determined primarily by its chemical species, with the inorganic species generally more harmful than the organic forms. In aqueous solutions, inorganic arsenic exists mainly as As(iii) and As(v).^5^ As(iii) normally acts as the dominant forms in natural water. Moreover, As(iii) is also more harmful and difficult to remove, compared to As(v).^6^ To remove the arsenic, several processes/technologies have been used, such as adsorption,^7^ precipitation,^8^ ion exchange,^9^ and membrane systems.^10^ Among them, adsorption is the most common method.^11^ However, As(iii) exists mainly as nonionic H_3_AsO_3_ when the pH value is less than 9, making its removal difficult by traditional adsorption methods. Hence, the pre-oxidation treatment of As(iii) to As(v) is a key step to decrease the toxicity and achieve higher arsenic removal rate *via* subsequent adsorption, precipitation, or filtration processes.^12^

In theory, oxidation of As(iii) can be realized using many chemical oxidants such as hydrogen peroxide, ozone, chlorine dioxide, chlorine, and potassium permanganate.^13–15^ However, despite the effective oxidation of As(iii), the use of chemical oxidants causes excess hazardous by-products and subsequent cleaning problems. To address these issues, several studies were made to remove arsenic from drinking water by means of photocatalytic oxidation for the merits of low power, easy handing, and high efficiency.^16–18^ The selection of photocatalyst is a key technology when using the photocatalytic method. Until now, TiO_2_ is the most common photocatalyst for arsenic removal in the reported literatures, benefit from its inexpensiveness, chemical stability, and nontoxicity.^17^ Nevertheless, due to its wide band gap (3.2 eV), the photocatalytic abilities of TiO_2_ can be excited only in ultraviolet region, meaning that most of solar power unable to use.^19^ Hence, its practical application in photocatalysts is limited. To obtain photocatalysts with excellent visible light response, expanding the absorption band of TiO_2_ from ultraviolet to visible light region is necessary. Various modification strategies were proposed to achieve this goal, such as structural design,^20^ metal or non-metal doping,^21,22^ heterojunction,^23^ and so on.^24,25^ However, most of these strategies were either complex or expensive.

Therefore, increasing attention has been paid to non-titania-based alternative catalysts in recent years, such as Bi_2_WO_6_,^26^ Ag_3_PO_4_,^27^ CdS,^28^ g-C_3_N_4_.^29^ Bismuth tungstate (BWO), a visible light induced semiconductor material with a low band-gap has shown excellent intrinsic physical and chemical properties including piezoelectricity, pyro-electricity, chemical stability, nontoxicity, and interesting optical properties.^30^ Nevertheless, like many other nanoparticle photocatalysts, the BWO photocatalyst easily becomes inactive or forms aggregates, and its reclamation is also difficult.^31^ To overcome these practical application problems, many researchers tried to design clay-based composite photocatalysts using natural clay minerals as support materials. It was found that many clay-based composite photocatalysts are extremely effective for degrading organic matter or detoxification of heavy metal, including attapulgite,^32^ zeolite,^33^ rectorite,^34^ bentonite,^35^ sepiolite,^36^ kaolinite.^37^ The improved activity of composite photocatalysts can be attributed to the fast reactant adsorption rate, enlarged surface area, and improved stability of the composite photocatalysts compared to the bare photocatalysts. Amongst the clay minerals, bentonite (BENT) is very attractive due to its abundance, porosity, chemical and mechanical stability, and non-toxic. So, BENT has been considered as a promising substrate for depositing photocatalysts, such as TiO_2_,^24^ In_2_O_3_,^38^ Cu_2_O,^39^ g-C_3_N_4_,^40^ and ZnFe_2_O_4_.^35^ However, as far as we know, removal of As(iii) through photocatalytic oxidation by BWO/BENT composites has never been reported. Thus, the present work aims to synthesize a novel kind of BWO/BENT composite photocatalysts and evaluate its performance in the photocatalytic removal of As(iii) under simulated sunlight irradiation. The structure, micromorphology, pore diameter, surface area, and photocatalytic performance of the BWO/BENT composites were obtained through systematic test and analysis. Based on the experiments, the mechanism of enhanced photocatalytic capability of BWO/BENT composites was also discussed. The study confirms that the BWO/BENT composites have the potential to be a good candidate for removal of As(iii).

## 2. Experimental

### 2.1 Materials and reagents

Natural BENT, obtained from Xinjiang Nonmetallic Minerals Xia Zi Jie Bentonite Co., Ltd. (Xin Jiang, China) has the main compounds of SiO_2_ 66.37%, Al_2_O_3_ 14.23%, Fe_2_O_3_ 5.07%, MgO 1.85%, Na_2_O 2.25%, CaO 0.81%, K_2_O 1.22%. Bi(NO_3_)_3_·5H_2_O, Na_2_WO_4_·2H_2_O, Na_2_HAsO_4_·7H_2_O, NaAsO_2_, (NH_4_)_6_Mo_7_O_24_·4H_2_O, C_6_H_8_O_6_, H_2_SO_4_, C_8_H_4_K_2_O_12_Sb_2_·3H_2_O, C_4_H_10_O (*t*-BuOH), C_6_H_4_O_2_ (BZQ), and C_10_H_14_N_2_Na_2_O_8_·2H_2_O (EDTA-2Na) were all provided by Sinopharm Chemical Reagents Co., Ltd. (Shanghai, China) and used without further treatment. The reserve solutions of As(v) or As(iii) with concentration of 1000 mg L^−1^ were prepared by dissolving Na_2_HAsO_4_·7H_2_O or NaAsO_2_ in ultrapure water. As(iii) reaction solutions were newly obtained by appropriate dilution of the corresponding stock solution with ultrapure water obtained from a Milli-Q water purification system (Millipore, USA).

### 2.2 Preparation of BWO/BENT nanocomposites

BWO/BENT composite photocatalysts were fabricated by hydrothermal method. Briefly, 2 mmol Bi(NO_3_)_3_·5H_2_O was mixed with 20 mL of 0.4 mol L^−1^ nitric acid to obtain a clear solution of bismuth nitrate. Next, 1 mmol Na_2_WO_4_·2H_2_O was added in 40 mL of ultrapure water to produce a transparent solution. Then, the two solutions and a certain proportion of BENT were mixed uniformly, and the pH value was regulated to 7. The resulting suspension was poured into a sealed Teflon-lined stainless steel autoclave with reaction volume of 100 mL, and kept at 180 °C for 24 h under autogenous pressure. Then the autoclave was cooled to ambient temperature. Finally, the resultant precipitates were separated by centrifugation, rinsed with ultrapure water and absolute ethanol repeatedly, and then followed by dried in vacuum drier at 70 °C to obtain the BWO/BENT products. As a contrast, pure BWO was synthesized using the same procedure but no BENT added.

### 2.3 Sample characterization

The phase structures of as-synthesized samples were analyzed by X-ray diffraction (X'Pert powder, PANalytical, Netherlands) with Cu Kα radiation (*λ* = 0.1546 nm). Surface morphology was examined by a scanning electron microscope (S-4800, Hitachi, Japan). The microstructure of prepared BWO/BENT-30% was analyzed using a transmission electron microscopy (JEOL JEM-2100F) operating at 200 kV. UV-vis diffuse reflectance spectra (DRS) were investigated using a UV-vis spectrophotometer (UV2550, Shimadzu, Japan), fitted with an integrating sphere accessory, and BaSO_4_ was adopted as the reflectance standard. The surface area of samples was conducted through N_2_ adsorption/desorption method on a BELSORP-mini II (MicrotracBEL, Japan). The X-ray photoelectron spectra (XPS) were obtained through a spectrometer (XPS, ESCALAB 250). Photoluminescence (PL) properties were measured on a RF-5301 PC spectrophotometer (Shimadzu, Japan) with emission range from 325 to 650 nm, and the exciting wavelength of 290 nm. The photocurrent characterization was measured on a CHI660C electrochemical workstation (Chenhua, China). Pt wire and saturated calomel electrode were employed as counter and reference electrode. The working electrode was the BWO/BENT samples spin-coated on an indium-tin oxide (ITO) glass (1 × 1.5 cm^2^), and Na_2_SO_4_ solution was selected as the electrolyte. A 300 W xenon lamp (Beijing Perfectlight Technology Co., Ltd, China) was the light source during the whole test process.

### 2.4 Photocatalytic activity tests

Photocatalytic activity of the obtained samples were analyzed in terms of removal rate of As(iii). A 300 W Xe lamp equipped with circulating cooling water was employed as the simulated sunlight source. The light irradiation was performed in a 250 mL cylindrical glass photoreactor, and the height between reaction liquid interface and light source was set as 10 cm. First, As(iii) solution (100 mL, 10 mg L^−1^) was added into the reactor, and 20 mg of as-synthesized catalysts photocatalyst was dispersed into it. Then, the obtained suspension was stirred constantly in the dark by a magnetic stirrer for 60 min to achieve adsorption/desorption equilibrium. For the As(iii) oxidation experiments, the mixed solution was exposed to the 300 W Xe lamp irradiation. At certain time intervals, 2.0 mL of the slurry was taken out and filtered using a 0.22 μm Millipore filter to remove solid impurity. The solution was subsequently analyzed to determine the individual arsenic species concentrations. To determine adsorption and photocatalytic properties of the synthesized photocatalysts, a series of experiments were performed with and without irradiation in the presence of photocatalysts, respectively. As a contrast, the solution of As(iii) was also treated just under simulated sunlight without the photocatalyst. *t*-BuOH, BZQ, and EDTA-2Na were chosen to clarify the active species under simulated sunlight irradiation.

### 2.5 Analytical measurements

Arsenic molybdenum blue method was employed to determine the As(v) concentration.^41^ Total arsenic concentration was detected by pre-oxidation treatment of As(iii) by KMnO_4_ (0.01 M). Thus, As(iii) in the solution was oxidized completely to As(v). So concentration of the arsenic solution for both before and after oxidation can be analyzed by molybdenum blue method. Finally, the concentrations of As(iii) was obtained by simple subtraction process.

## 3. Results and discussion

### 3.1 XRD analysis

**Fig. 1:**
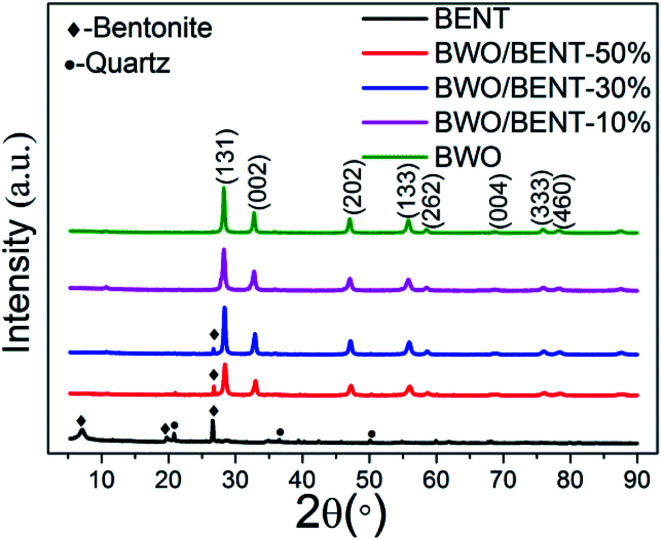
XRD patterns of BWO/BENT samples with different mass ratios.

XRD patterns of the BWO, the pristine BENT, and the BWO/BENT composites prepared with different proportions are shown in Fig. 1. The reflections at 2*θ* values of 19.86°, 26.67°, and 27.59° were attributed to the peaks of BENT (JCPDS card no. 00-013-0259). The other peaks present at 18.61°, 19.80°, and 26.55° were due to quartz (JCPDS card no. 01-079-1910) and other impurities in BENT. For the prepared BWO particles, the XRD patterns showed reflections at the 2*θ* values of 28.3°, 32.8°, 47.1°, 55.8°, 58.5°, 68.7°, 76.1°, and 78.5°, which were indexed as the (131), (002), (202), (133), (262), (004), (333), and (460) reflections of pure BWO, respectively (JCPDS card no. 00-039-0256). The decreased intensity of the (001) XRD peak of BENT in the composite samples meant that the structure of BENT may be destructed in some degree. Except for the case with a small amount of BENT, nearly all the reflections of BWO/BENT composites could be assigned to orthorhombic BWO, indicating the successful formation of BWO/BENT composites.

### 3.2 SEM and TEM images

**Fig. 2:**
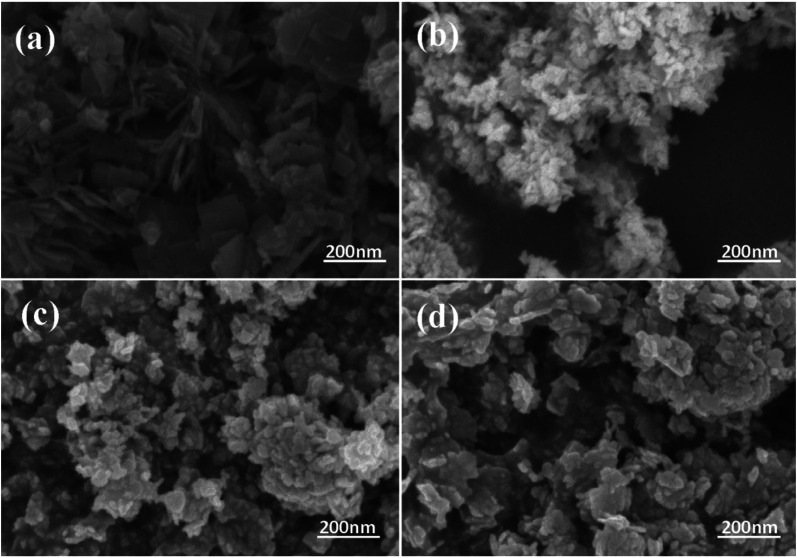
SEM images of BWO (a), BWO/BENT-10% (b), BWO/BENT-30% (c), and BWO/BENT-50% (d) samples.

**Fig. 3:**
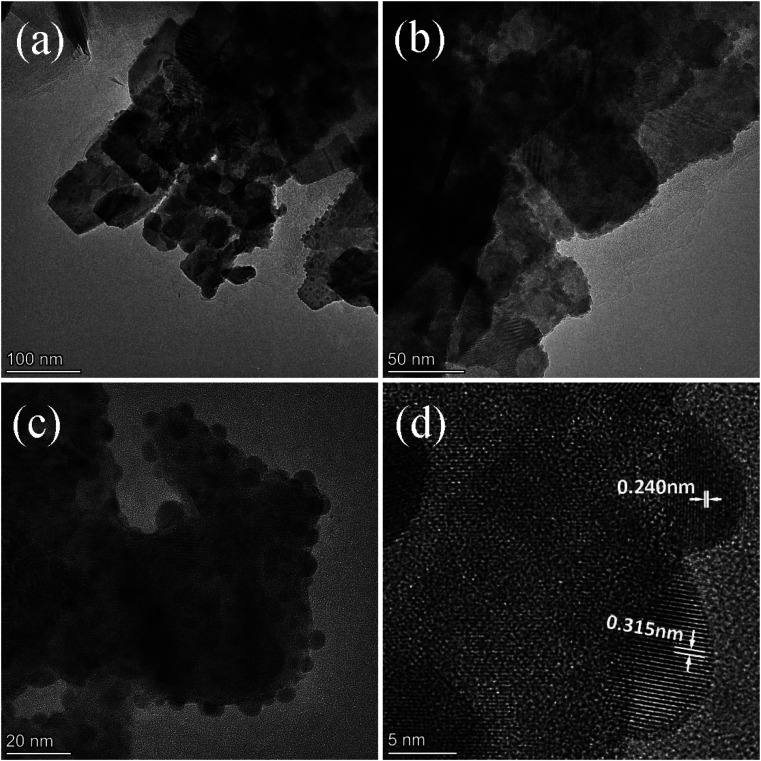
TEM (a–c) and HRTEM (d) images of BWO/BENT-30%.

Fig. 2 exhibits the morphology of the pure BWO and composite catalysts. BWO appeared with a regular square flake structure, with a relatively uniform width and thickness (Fig. 2a). Meanwhile, compared to pure BWO, the BWO/BENT composites had a sheet-like structure due to the layered BWO and BENT clay. Moreover, the ratio of aggregates became less when the loading of BENT increased from 10% to 50% (Fig. 2b–d). The microstructure of BWO/BENT-30% was deep studied through TEM and HRTEM analyses. The results were shown in Fig. 3a–c, indicating that small particles had diameter of about 5 nm and uniformly dispersed on the surface of bentonite, which is coincided with the SEM results. HRTEM image reveals that the lattice fringes spaced by 0.315 and 0.240 nm were observed, corresponding to the lattice spacing of (113) and (121) planes of orthorhombic Bi_2_WO_6_, respectively. From these images, it was clearly observed that the BWO nanoparticles were well dispersed over the BENT clay, reflecting the good combination between them and also proving the existence of BWO/BENT nanocomposites, in agreement with the XRD analysis.

### 3.3 UV-vis diffuse reflectance spectra

**Fig. 4:**
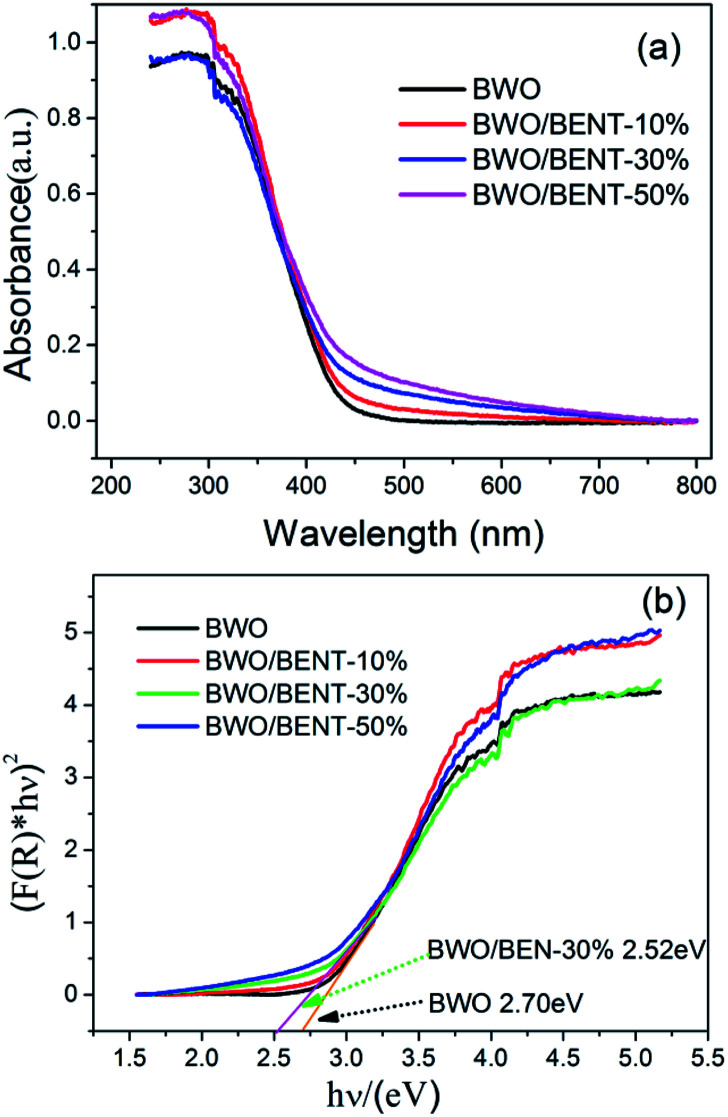
(a) UV-vis diffuse reflectance spectra of BWO/BENT samples with different mass ratios; (b) estimated band gaps of BWO/BENT-30% composite.

The UV-vis diffuse reflection spectra of the BWO/BENT composites with different mass ratios are shown in Fig. 4. Compared to pure BWO, the BWO/BENT composite catalysts showed an improved light absorption range and higher intensity in the whole spectral region. The absorption edges of BWO/BENT composites were found to be more than 460 nm showing a slight red shift due to cooperation of the two materials. This red shift meant better photocatalytic performance and lower band gap energy than that of pure BWO.

Moreover, the Kubelka–Munk function was employed to estimate the band gap of BWO and BWO/BENT-30%,^42^ and the values calculated from the plot of (*F*(*R*) × *hν*)^2^*versus hν* are 2.70 and 2.52 eV, respectively. This result confirmed that the BENT support could broaden the photo-absorption region and decrease the band gap of BWO, which might help improve the ability of photocatalysis oxidation reaction.

### 3.4 N_2_ adsorption/desorption

**Fig. 5:**
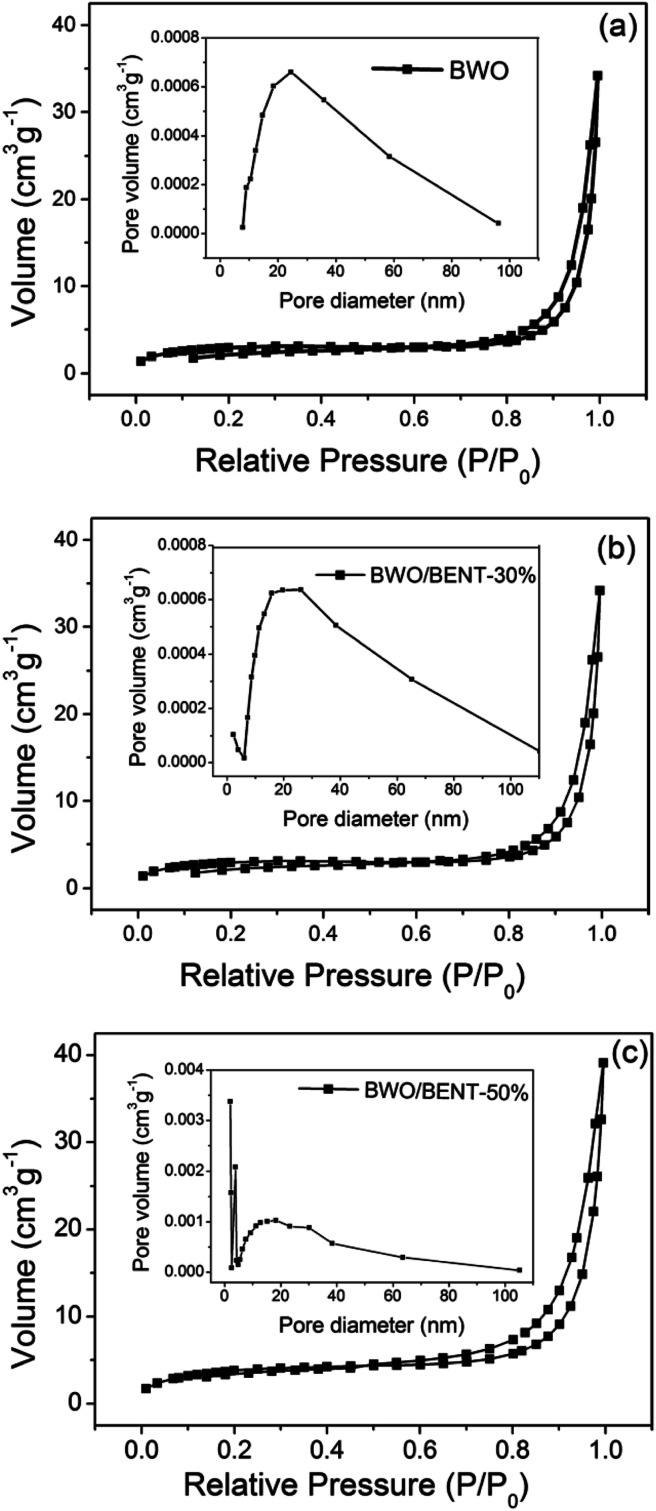
N_2_ adsorption–desorption isotherm and pore size distribution (inset) of BWO/BENT samples with different mass ratios: BWO (a), BWO/BENT-30% (b), and BWO/BENT-50% (c).

The N_2_ adsorption/desorption results were shown in Fig. 5. All three samples exhibited reversible type IV isotherm with hysteresis loops corresponding to type H3, implying mesoporous characteristics of the samples. The corresponding pore size distribution (inset) obtained through the BJH method showed that the pore diameters of these samples ranged from 2 to 50 nm. As shown in Table 1, the BET surface areas were measured to be 8.94, 10.59, and 14.03 m^2^ g^−1^ for BWO, BWO/BENT-30%, and BWO/BENT-50%, respectively. The BET surface area of the composites generally increased with the BENT content increasing. The high specific area may promote adsorption and provide more reactive sites, which are favorable for achieving high photocatalytic activities.

**Table 1:** BET surface area *S*_BET_, average diameter *d*_a_, and total pore volume *V*_t_ of BWO/BENT samples with different mass ratios

Sample	*S*_BET_ (m^2^ g^−1^)	*d*_a_ (nm)	*V*_t_ (cm^3^ g^−1^)
BWO	8.94	50.24	0.04
BWO/BENT-30%	10.59	38.48	0.051
BWO/BENT-50%	14.03	26.91	0.059

### 3.5 Photocatalytic oxidation performance

**Fig. 6:**
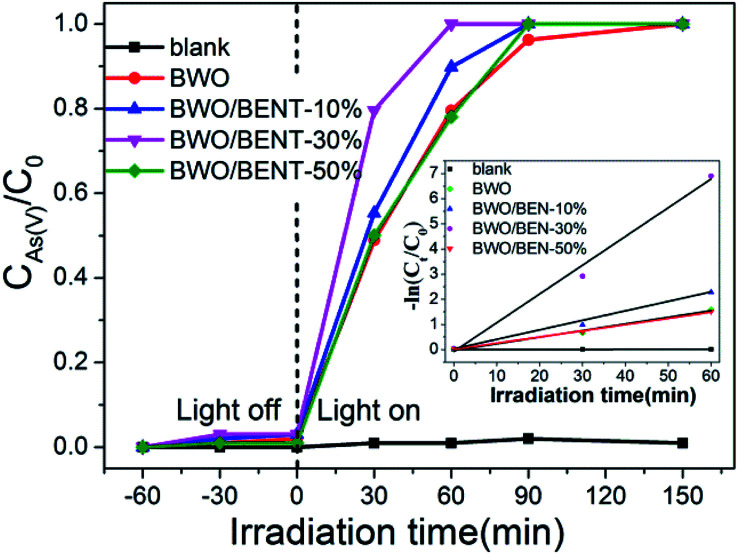
Photo-oxidation activities of As(iii) by BWO and BWO/BENT composites.

To evaluate potential applicability of the prepared BWO/BENT nanocomposites, the efficiencies of pure BWO and BWO/BENT with different ratios were separately evaluated in the oxidation of As(iii). The photocatalyst dosage was 0.2 g L^−1^ and the initial arsenic concentration was 10 mg L^−1^. As shown in Fig. 6, during the dark adsorption process, all the prepared catalysts showed poor adsorption for As(iii), and the photocatalytic oxidation of As(iii) without the photocatalyst was almost negligible.

After the Xe lamp was turned on, all samples showed the ability to oxidize As(iii). For pure BWO, as much as 79.58% of the As(iii) was oxidized after 1 h of irradiation. In comparison, all BWO/BENT samples exhibited even better performance. The photocatalytic oxidation ability increased remarkably as the content of BENT in the composites increased from 10% to 30%, and then decreased at higher BENT content. So, the content of BENT clearly had a great influence on the As(iii) removal efficiency. A low amount of BENT was beneficial to promote the photo-oxidation of As(iii). However, when its mass ratio was too high (*e.g.* 50%), the photocatalytic activity for As(iii) oxidation was decreased, which was attributed to the decreased content of BWO. Moreover, among the as-synthesized photocatalysts, the BWO/BENT-30% composite exhibited the highest photocatalytic activity, and the As(iii) was almost completely oxidized to As(v) after 60 min of illumination. Due to the low concentration of the arsenic solution (10 mg L^−1^), the photo-oxidation of As(iii) conforms to pseudo-first-order kinetics model ln(*C*_0_/*C*_*t*_) = *kt*, where *k* is the apparent reaction rate constant, *C*_0_ and *C*_*t*_ are the concentrations of As(iii) at the irradiation time 0 and *t*, respectively.^43^ As shown in the inset of Fig. 6, the calculated As(iii) oxidation rate constants (*k*) of were 0.00017, 0.02614, 0.03756, 0.0878 and 0.02511 min^−1^ for blank, BWO, BWO/BENT-10%, BWO/BENT-30% and BWO/BENT-50%, respectively. Compared with pure BWO, the catalytic performance of BWO/BENT-30% was improved by more than 3 times. Therefore, the modification of BWO with BENT could improve the photo-oxidation activity of As(iii). The increased As(iii) removal efficiency might benefit from the enlarged surface area, the enhanced optical response, the reduced agglomeration of BWO nanoparticles. The BENT acted as a support material to improving the dispersion of BWO nanoparticles and preventing the particle agglomeration. Overall, the comparative study showed that the as-synthesized BWO/BENT nanocomposites could be employed as a new efficient photocatalyst for removing the As(iii) under simulated sunlight irradiation.

**Fig. 7:**
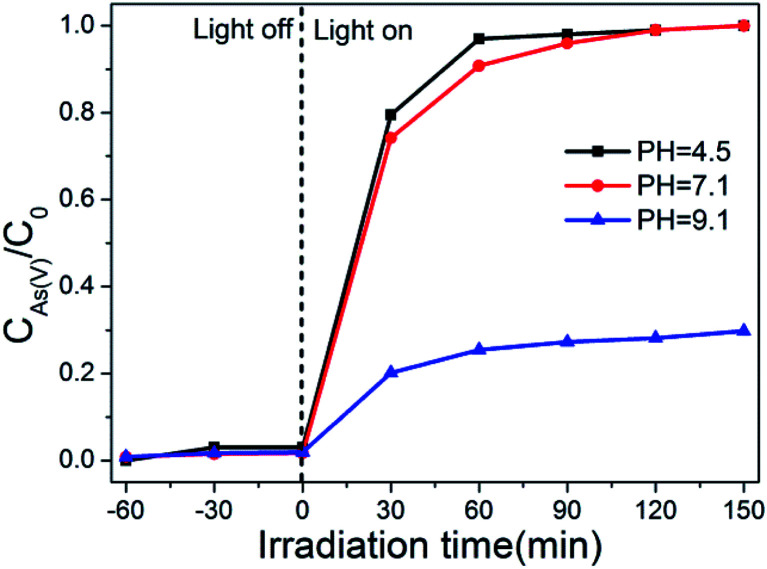
Effect of initial solution pH on the photo-oxidation of As(iii) by BWO/BENT-30% composite.

The effect of pH values on the photo-oxidation of As(iii) over the optimal composite photocatalyst (BWO/BENT-30%) was investigated. From Fig. 7, within the studied pH range, almost complete oxidation of As(iii) was achieved in neutral and alkaline conditions (pH > 7.1) after placed under simulated sunlight irradiation for 90 min. However, when the initial pH decreased to 4.5, the oxidation of As(iii) was significantly inhibited, possibly due to the low stability of BWO under acidic conditions.

**Fig. 8:**
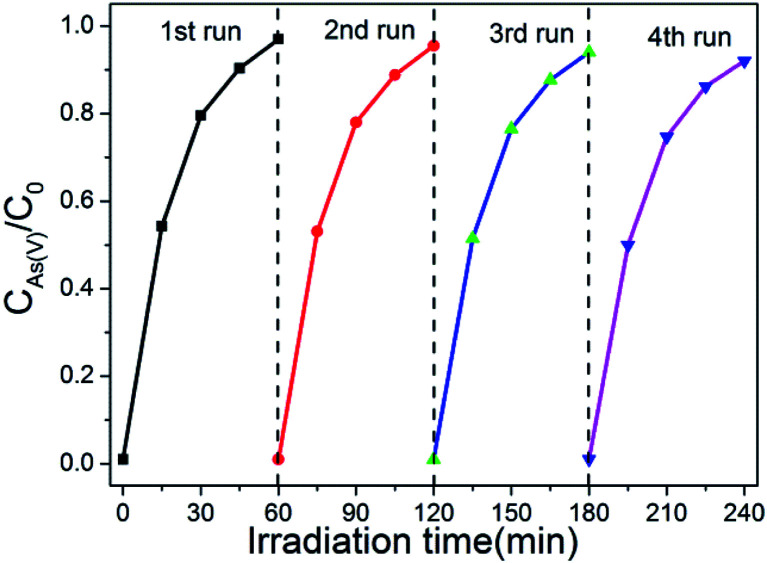
Photo-oxidation of As(iii) over recycled BWO/BENT-30%.

The recyclability of photocatalysts is also a important factor for its application. In order to confirm the stability of as-synthesized photocatalyst, the cycling tests of the photo-oxidation of As(iii) in the case of existing of BWO/BENT-30% were performed. As shown in Fig. 8, four cycles of photo-oxidation of the As(iii) experiment were carried out at the same operation conditions. After each experiment, the photocatalyst was regained by centrifugation method and continued to use in next test cycle. It is shown that the oxidation effect of As(iii) by BWO/BENT-30% did not change obviously after 4 cycles, indicating high stability and excellent reusability during the photo-reaction.

### 3.6 Surface characterization using XPS

**Fig. 9:**
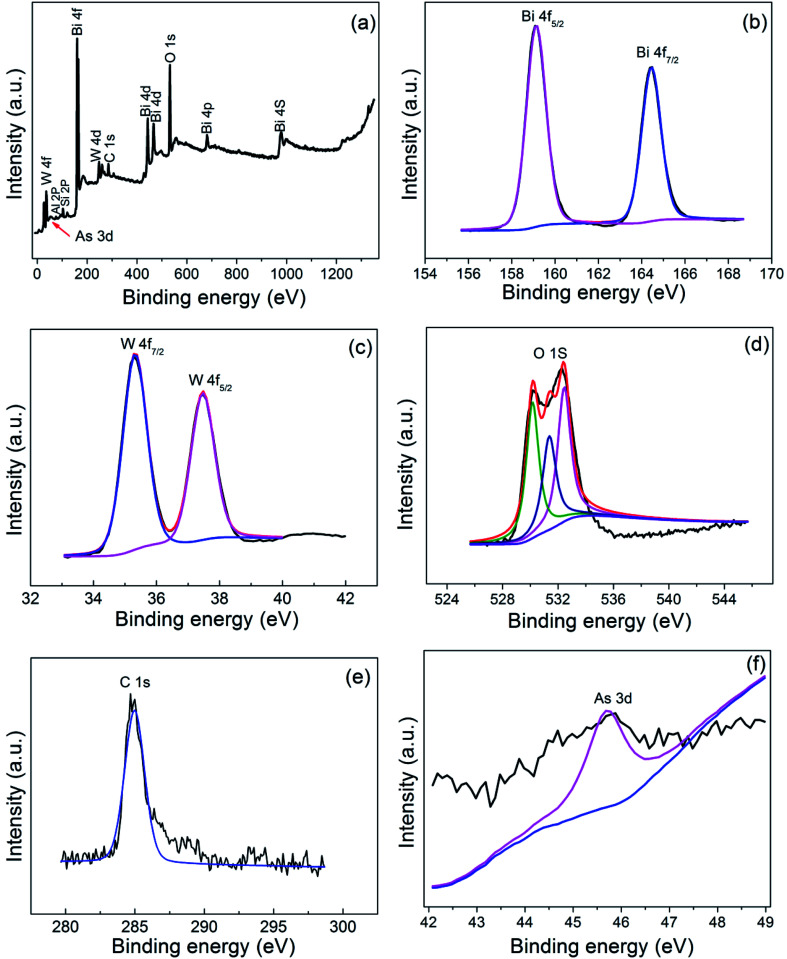
XPS spectra of BWO/BENT-30%: (a) survey, (b) Bi 4f, (c) W 4f, (d) O 1s, (e) C 1s, and (f) As 3d.

Fig. 9 showed the XPS spectra of the BWO/BENT-30% photocatalyst treated in the As(iii) solution under simulated sunlight irradiation. The full survey spectrum in Fig. 9a exhibited that Bi, W, O, C, Al, Si, and As existed in the photocatalyst. The C peak may attribute to the impurities or adventitious carbon present in the samples or in the XPS instrument, and Al, Si originate from the bentonite. For accurate analysis, high resolution spectrum of the Bi 4f, W 4f, O 1s, C 1s, As 3d were also provided. In Fig. 9b, the binding energy at 164.4 eV and 159.1 eV can be assigned to Bi 4f_7/2_ and Bi 4f_5/2_, respectively, indicating a Bi^3+^ chemical state in the composite.^44^ The W 4f XPS spectra of the sample was shown in Fig. 9c, exhibiting W 4f_7/2_ and W 4f_5/2_ peaks at 35.5 and 37.6 eV, corresponding to W^6+^ species.^45^ The O 1s spectra of the samples (Fig. 9d) present three peaks, corresponding to the lattice oxygen in Bi–O (529.7 eV), W–O (531.2 eV), and surface adsorbed oxygen (532.6 eV).^46^ The As 3d XPS spectra was shown in Fig. 9e, the peak at 45.6 eV can be ascribed to As(v).^47^ However, the peak at 45.6 eV was very weak, suggesting that only a very small amount of arsenic was adsorbed on the photocatalyst, which is coincident with the photocatalytic experiment results.

### 3.7 PL and photocurrent analysis

**Fig. 10:**
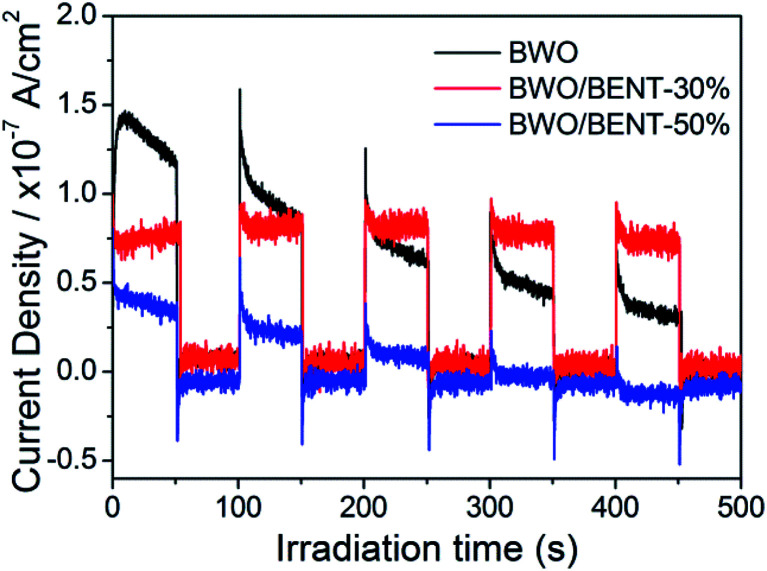
Periodic on/off photocurrent responses for BWO and BWO/BENT composites.

Fig. 10 shows the measured surface photocurrents under Xe lamp illumination. For pure BWO, the photocurrent intensity decreases with irradiation time, this experimental phenomenon is similar to previous research.^48,49^ The reason may be due to the fact that photo-generated electrons in the inner layer of Bi_2_WO_6_ need to overcome a long migration distance to reach the surface, and electrons can accumulate and recombine with holes during the irradiation time.^50^ However, the photocurrent of BWO/BENT-30% kept constant with time and was higher than those of pure BWO and BWO/BENT-50%. This may be due to that the agglomeration of BWO nanoparticles is suppressed through introduction of appropriate amount of BENT, which helps to form monolayer BWO and improve the electron–hole pair separation.

**Fig. 11:**
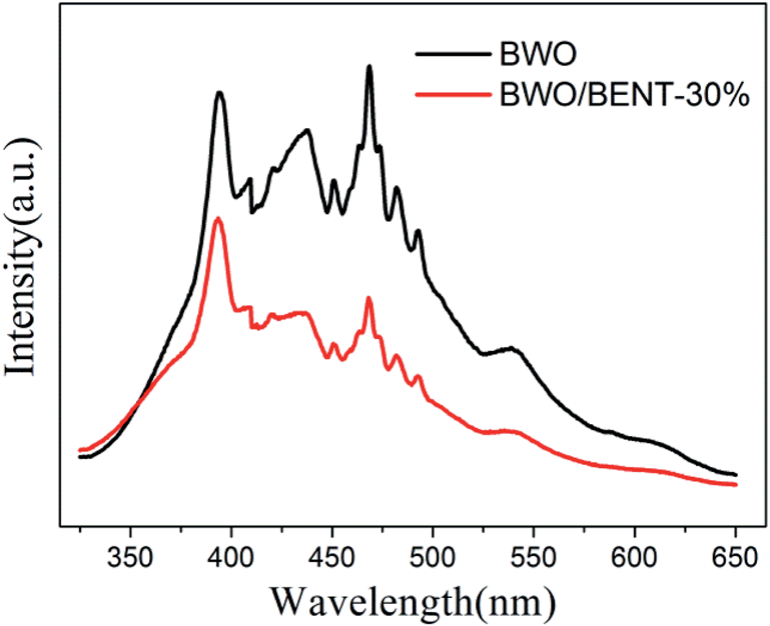
PL spectra of BWO and BWO/BENT-30% composite.

Photoluminescence (PL) is an effective method to investigate separation effect of electrons and holes produced over photocatalysts. Generally, a higher PL intensity indicates faster recombination of the charge carriers, *i.e.*, a lower photocatalytic activity. Fig. 11 presents the PL spectra of BWO and BWO/BENT-30% composite obtained at an excitation wavelength of 290 nm. The PL intensity of BWO/BENT-30% composite was much lower than that of pure BWO, indicating a reduced electron–hole recombination rate due to the introduction of BENT. This improved separation performance may be caused by the synergistic effect of BWO and BENT. This finding is consistent with the result of photocurrent test.

### 3.8 Active species identification

**Fig. 12:**
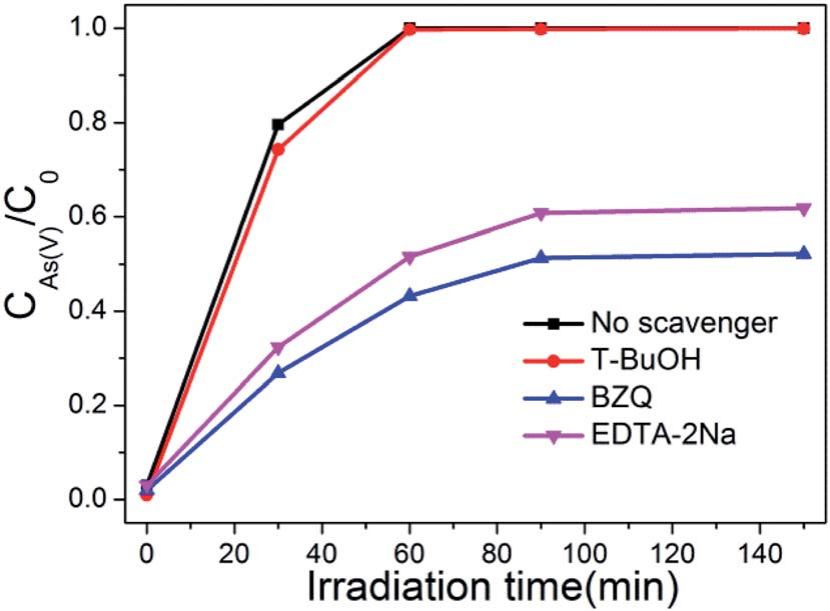
Effect of the scavengers on As(iii) oxidation in the presence of BWO/BENT-30% composite.

It is generally known that photocatalyst can be activated by light with suitable wavelength to generate electrons and holes, then form free radicals such as hydroxyl radical (·OH), superoxide radical (·O_2_^−^), and hole (h^+^). These free radicals have a strong capability to effectively oxidize various pollutants.^51^ To verify the dominant active species in the photocatalytic oxidation process of As(iii), free radical quenching experiments were conducted using different scavengers. *t*-BuOH, BZQ and EDTA-2Na were chosen to scavenge the ·OH, ·O_2_^−^ and h^+^, respectively. As show in Fig. 12, when no scavenger was added, BWO/BENT-30% could oxidize nearly 100% of the arsenic within 60 min. The addition of *t*-BuOH, a ·OH scavenger, did not obviously suppressed the As(iii) oxidation. However, when BZQ or EDTA-2Na was used, an obvious suppression in the oxidation of As(iii) was observed. Therefore, both ·O_2_^−^ and h^+^ radicals were confirmed as the major contributors for photocatalytic oxidation of As(iii) by BWO/BENT-30% composite under simulated sunlight irradiation. In contrast, ·OH did not directly contribute to the oxidation of As(iii) during the photocatalytic process.

### 3.9 Photocatalytic mechanism

**Fig. 13:**
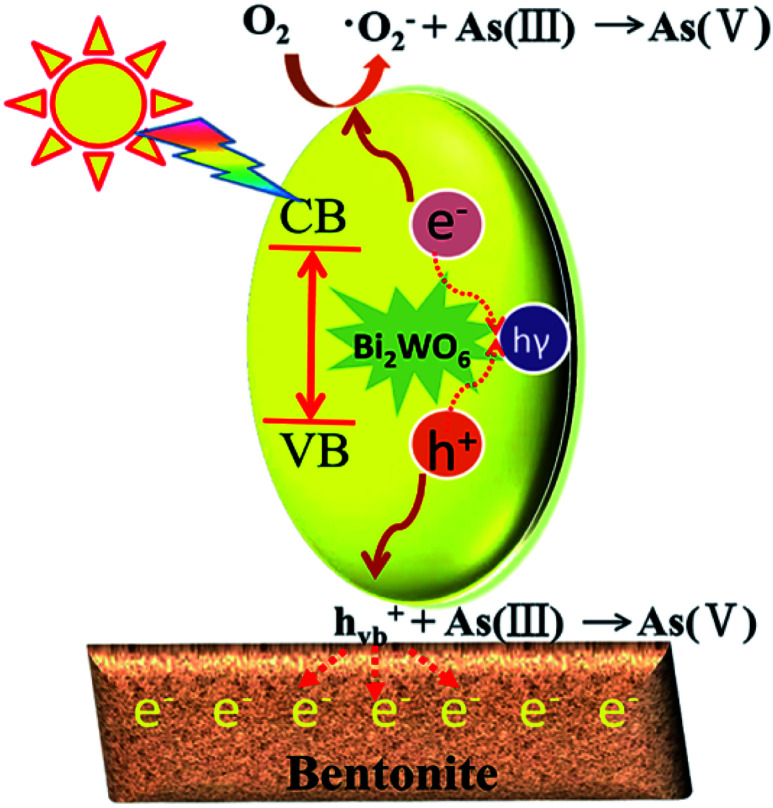
Photocatalytic mechanism of BWO/BENT composites under simulated sunlight illumination.

According to the above analysis, several reasons may be responsible for the improved photocatalytic ability of the prepared composite photocatalysts. Firstly, the absorption edge of BWO/BENT-30% showed a slight red shift in the UV-vis diffuse reflectance tests, which indicated that the composite material had better photocatalytic response ability and lower band gap energy than those of the pure BWO. Second, BWO/BENT-30% exhibited a larger BET surface area, meaning that more active sites were available for the photo-oxidation of As(iii). Third, SEM analysis showed that the BENT support can reduce the agglomeration of BWO particles, which help improve the photocatalytic ability. Fourth, because of the electrostatic attraction between the positively charged hole and the negatively charged BENT, the recombination of electrons and holes could be inhibited effectively. Hence, the activity of the composite photocatalyst was enhanced.^52^ Based on the analysis described above, a plausible process for the photo-oxidation of As(iii) over BWO/BENT composite was proposed as follows (Fig. 13 and eqn (1)–(4)). (a) When illuminated with simulated sunlight, valence band (VB) electrons (e^−^) of BWO can be activated by the high energy photons (*hν*) and transfer to the conduction band (CB), simultaneously producing holes (h^+^) in the VB according to eqn (1). (b) Compared with redox potential of O_2_/·O_2_^−^ (−0.33 eV), the conduction band (CB) potential of BWO is more negative (−0.84 eV),^53^ so the dissolved O_2_ could be reduced to ·O_2_^−^ radicals, which are the important active species in the oxidation of As(iii) (eqn (2)). (c) Moreover, as the VB potential (*E*_VB_) of BWO (1.89 eV) was more positive than that of As(iii)/As(v) (*E*_As(III)/As(V)_ = 0.56 eV *vs.* NHE),^53,54^ As(iii) can be directly oxidized by the photo-generated holes. Hence, the main active species were identified to be ·O_2_^−^ and h^+^ responsible for the As(iii) conversion during the photocatalytic process (eqn (3) and (4)). The reaction could be described by the following reaction routes:

1 BWO + *hν* → BWO (e_cb_^−^) + BWO (h_vb_^+^)

2 BWO (e_cb_^−^) + O_2_ → BWO + ·O_2_^−^

3 As(iii) + ·O_2_^−^ → As(v)

4 As(iii) + h^+^ → As(v)

## 4. Conclusions

BWO/BENT composites were successfully synthesized at 180 °C by a hydrothermal method. Compared to pure BWO, the absorption edge of the BWO/BENT composite catalysts exhibited a slight red shift. As the mass percentage of BENT increases, the BET surface area of BWO/BENT composite also increases. More notably, the BWO/BENT composites exhibited better photocatalytic ability than pure BWO for the oxidation of As(iii) when irradiated under the simulated sunlight. Such a synergistic effect could be benefit from increased surface area, reduced aggregation, and enhanced electron–hole separation efficiency. Meanwhile, among the different BWO/BENT proportions, the BWO/BENT-30% composite shows the highest activity for the oxidation of As(iii). Radical quenching experiment confirm that the ·O_2_^−^ and h^+^ are the main active species responsible for the oxidation of As(iii). This study paves a novel pathway for the application of clay-based materials into environmental remediation.

## Conflicts of interest

There are no conflicts to declare.

## Supplementary Material
