# Correction: Synthesis of Bi_2_WO_6_/Na-bentonite composites for photocatalytic oxidation of arsenic(iii) under simulated sunlight

**DOI:** 10.1039/c9ra90092f

**Published:** 2019-12-09

**Authors:** Quancheng Yang, Yunxiang Dai, Zijian Huang, Jing Zhang, Ming Zeng, Changsheng Shi

**Affiliations:** School of Chemical and Environmental Engineering, China University of Mining and Technology Beijing 100083 P. R. China; Key Laboratory of Environmental Nano-Technology and Health Effect, Research Center for Eco-Environmental Sciences, Chinese Academy of Sciences Beijing 100085 P. R. China jingzhang@rcees.ac.cn; National Engineering Laboratory for VOCs Pollution Control Materials & Technology, University of Chinese Academy of Sciences Beijing 101408 P. R. China; Department of Environmental Engineering, North China Institute of Science and Technology Beijing 101601 P. R. China northinstitute@yeah.net

## Abstract

Correction for ‘Synthesis of Bi_2_WO_6_/Na-bentonite composites for photocatalytic oxidation of arsenic(iii) under simulated sunlight’ by Quancheng Yang *et al.*, *RSC Adv.*, 2019, **9**, 29689–29698.

Ref. 28 in the published article was incorrect, with an incorrect page range provided. The correct version is shown as ref. 1 below.

The Royal Society of Chemistry apologises for these errors and any consequent inconvenience to authors and readers.

## Supplementary Material
